# Phonon anharmonicities and ultrafast dynamics in epitaxial Sb_2_Te_3_

**DOI:** 10.1038/s41598-020-69663-y

**Published:** 2020-07-31

**Authors:** V. Bragaglia, M. Ramsteiner, D. Schick, J. E. Boschker, R. Mitzner, R. Calarco, K. Holldack

**Affiliations:** 1 Paul-Drude-Institut für Festkörperelektronik, Leibniz-Instiut im Forschungsverbund Berlin e. V., Hausvogteiplatz 5-7, 10117 Berlin, Germany; 2 Helmholtz-Zentrum Berlin für Materialien und Energie GmbH, Albert-Einstein-Str. 15, 12489 Berlin, Germany; 3 Max-Born-Institut für Nichtlineare Optik und Kurzzeitspektroskopie, Max-Born-Straße 2A, 12489 Berlin, Germany; 4 Istituto per la Microelettronica E Microsistemi (IMM), Consiglio Nazionale delle Ricerche, Via del Fosso del Cavaliere 100, 00133 Rome, Italy; 5 Present Address: IBM Research-Zürich, Säumerstrasse 4, 8803 Rüschlikon, Switzerland

**Keywords:** Raman spectroscopy, Electronic properties and materials, Two-dimensional materials, Condensed-matter physics, Techniques and instrumentation, Physics, Terahertz optics, Ultrafast photonics

## Abstract

In this study we report on the investigation of epitaxially grown Sb_2_Te_3_ by employing Fourier-Transform transmission Spectroscopy (FTS) with laser-induced Coherent Synchrotron Radiation (CSR) in the Terahertz (THz) spectral range. Static spectra in the range between 20 and 120 cm^−1^ highlight a peculiar softening of an in-plane IR-active phonon mode upon temperature decrease, as opposed to all Raman active modes which instead show a hardening upon temperature decrease in the same energy range. The phonon mode softening is found to be accompanied by an increase of free carrier concentration. A strong coupling of the two systems (free carriers and phonons) is observed and further evidenced by exciting the same phonon mode at 62 cm^−1^ within an ultrafast pump-probe scheme employing a femtosecond laser as pump and a CSR single cycle THz pulse as probe. Separation of the free carrier contribution and the phonon resonance in the investigated THz range reveals that, both damping of the phonon mode and relaxation of hot carriers in the time domain happen on the same time scale of 5 ps. This relaxation is about a factor of 10 slower than expected from the Lorentz time-bandwidth limit. The results are discussed in the framework of phonon scattering at thermal and laser induced transient free carriers.

## Introduction

Electronic and lattice dynamics in chalcogenide-based materials are important factors in the performance of opto-electrical data-storage media and thermoelectric devices. Among these materials, Sb_2_Te_3_ is a prototype as it is, together with GeTe and BiTe, one of the constituents of chalcogenide superlattices (CSLs)^1,2^. Combining Sb_2_Te_3_ with one of the above-mentioned crystals together in one lattice, creates a superlattice with intriguing properties different or greater than those of its individual components. In a recent study it has been demonstrated that a strong link exists among structural and thermoelectric properties in epitaxial Sb_2+*x*_Te_3_, the latter having implications also in phase change SLs upon strain engineering in designed Sb_2+*x*_Te_3_/GeTe multilayers^1^. Improved thermoelectric properties by nanostructuring Sb_2_Te_3_/BiTe multilayer have already been demonstrated^3^. Some more interesting properties include ultra-low power (non-melting) phase-change switching if compared to the established devices based on single active phase change material (PCM) and controlled topological states upon stack engineering^4–6^. Those properties can be exploited for applications such as in photonic and electronic memories and processors, in Terahertz (THz) detection and to regenerate electricity from waste heat. However, the electro-optic properties of these chalcogenide topological insulators have barely been explored^7,8^. The THz regime results ideal for the understanding of fundamental properties in such class of materials, as also shown by our previous works on GeSbTe alloys^9,10^.

Such measurement capability combined to a CSLs engineering could show future avenues for device improvements. To this purpose, in this work we present a dedicated study in the THz range of the Sb_2_Te_3_ constituent which is paramount for the study of CSLs dielectric properties and its carrier and lattice dynamics upon laser excitation.

Sb_2_Te_3_ is a narrow-band-gap semiconductor (E_g_ = 0.26 eV around room temperature) with *R*-3* m* space group and is composed of repeated planes of five-atomic layer lamellas separated by a van der Waals gap^11^. It exhibits a rather large concentration of p-type carriers around 10^20^ cm^−3^ ^1^ due to the presence of native antisite defects–Sb atoms occupying Te lattice sites–and is diamagnetic.

In literature, lattice dynamics and infrared-active lattice vibrations of in the rhombohedral V_2_–VI_3_ compounds Bi_2_Te_3_, Bi_2_Se_3_ and Sb_2_Te_3_ can be found^12,13^. Those, together with their thermal conductivity investigation of Bi_2_Te_3_ and Bi_2_Se_3_ as a function of various doping materials, revealed the influence of considerable anharmonic effects for this class of materials.

However, the fundamental understanding of carrier behavior in Sb_2_Te_3_ remains controversial, being affected by the contribution of topological and bulk states to the carrier behavior and by the structural properties of the material. Both can vary depending on the fabrication technique^14–16^. The work is organized into a first part in which static THz spectroscopy is used to probe an in-plane phonon mode at equilibrium upon changing temperature. In a second part instead, the same phonon mode is probed dynamically on an ultrafast timescale within a pump-probe scheme employing a femtosecond laser as pump and the THz light as probe. Results are discussed in the framework of strong electron-phonon interaction and phonon mode damping by hot free carriers.

## Results and discussion

### Part 1: Temperature dependent measurements

**Figure 1:**
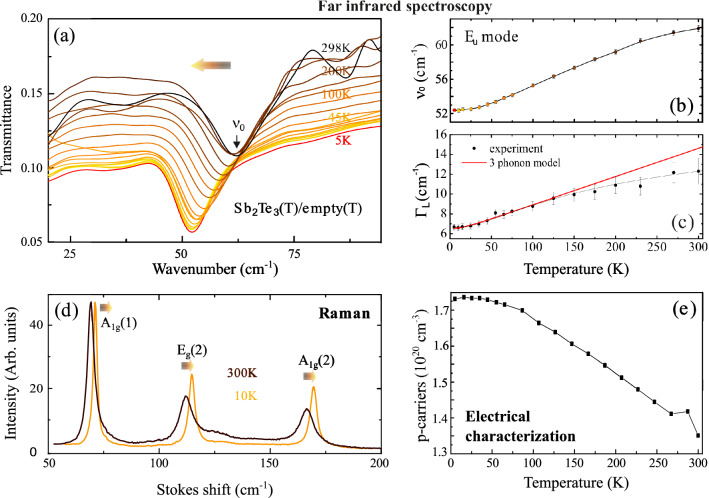
**(a)** Temperature dependent THz transmittance change of Sb_2_Te_3_. Curves are normalized to the empty case with a baseline becoming more noisy at around 300 K. **(b)** E_u_ phonon mode softening upon temperature decrease. **(c)** Experimental change (squares) of the peak width (FWHM) and fit (red line) as described in the text. **(d)** Raman spectra of the same Sb_2_Te_3_ sample measured at 300 K and 10 K with labeled modes^1,6^. Hardening of all modes is observed as T is decreased, as opposed to the IR active mode in **(b)**. **(e)** Electrical measurement of free carrier increase upon temperature change. The error on the carrier measurement is < 4%. Color code corresponding to the different temperatures is reported in the legend in **(a)**. Colored graded arrows indicate mode softening for the IR-active mode and mode hardening for all Raman modes upon temperature decrease, respectively.

**Figure 2:**
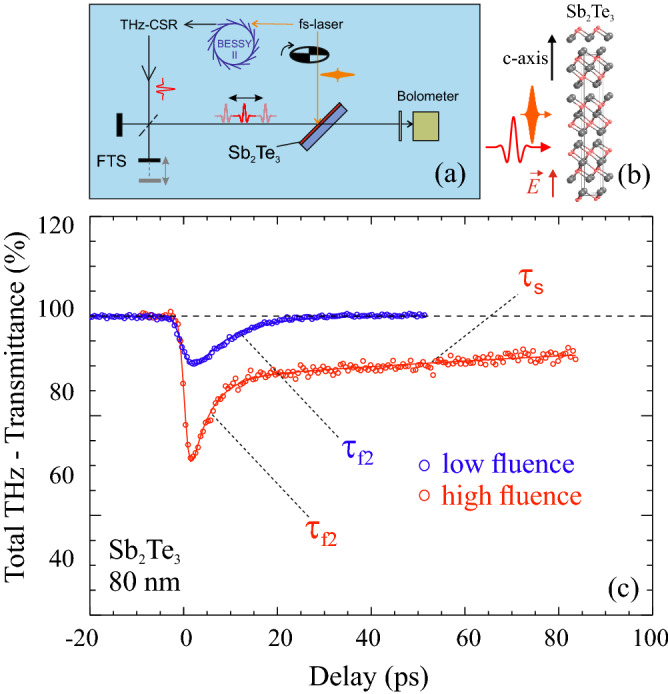
**(a)** Sketch of the ultrafast THz-FTS setup illustrating the working principle of spectral mapping of transient THz absorption in thin films employing single cycle coherent THz pulses from BESSY II. **(b)** Structure model of Sb_2_Te_3_ and marked directions of the electric field direction with respect to the c-axis. **(c)** Time traces of the total THz transmittance change in 80 nm epitaxial Sb_2_Te_3_ films upon 800 nm fs-laser excitation with different fluences (low fluence = 0.6 mJ/cm^2^, high fluence = 3.0 mJ/cm^2^).

We report here on temperature dependent measurements of Sb_2_Te_3_ far-infrared spectra acquired in transmittance configuration over a broad range of frequencies (20 to 90 cm^−1^) and temperatures (300 K down to 5 K). The spectra are normalized to the spectra taken without sample at the same temperature (T) (“empty case”). The semi-insulating Silicon substrate’s (R > 5 kΩ cm) transmission change can be neglected in this range of T, as found by reference measurements (not shown). Transmission spectra, in general, reflect the two contributions of free-carrier and phonon absorption. In this study the spectra were collected with the THz electric field vector oriented parallel to the [111] oriented c-axis (see Fig. 2b) to probe predominantly the in-plane charge dynamics. The results are shown in Fig. 1a. The absorption feature centered at 62 cm^−1^ (300 K) is attributed to the transverse optical (TO) IR-active E_u_ mode of Sb_2_Te_3_, vibrating perpendicularly to the c-axes, according to Richter et al.^11^ Note that in our previous study^9^ it has been erroneously attributed to an IR active A_u_ mode. As temperature is decreased, a total decrease of THz transmittance is observed (~ 33%), phenomenologically attributed to an increase of conductivity due to a raise of free carrier concentration and/or increase of carrier mobility. The temperature-dependent carrier concentration in the same sample, obtained by Hall measurement, is shown in Fig. 1e. Indeed, an increase of the free carrier concentration upon decreasing T is observed which is typical for Sb_2_Te_3_, as reported in previous studies^17^. Moreover, recent density functional theory (DFT) calculations show that the band gap of Sb_2_Te_3_ increases with increasing temperature due to the thermal expansion of Sb_2_Te_3_^18^ and such raise of the bandgap would result in a reduction of the free carrier concentration. Experimental verification of the theory can be found in Boschker et al.^19^.

Interestingly, as temperature is decreased the frequency position (ν_0_) of the peak progressively shifts toward lower wavenumbers from ν_0_ = 62 cm^−1^ (300 K) to ν_0_ = 52 cm^−1^ (5 K), as shown in Fig. 1b. However, the peak position change tends to saturate below 20 K and becomes less pronounced close to 300 K. The slight asymmetric line shape of the dip at 62 cm^−1^ evolving upon temperature (Fig. 1a) was fully reproduced by the Drude–Lorentz simulation of the film transmission (see also Fig. S1).

The interpretation of the transmittance in the THz range is not trivial for Sb_2_Te_3_ and similar materials as also reported in literature^11^, since the contribution to both reflectivity and transmittance measurements stem from both free carriers and phonons, and the decoupling of the two contributions is not straightforward. However, for Sb_2_Te_3_ a strong phonon resonance can be well separated from the relatively broad spectral feature induced by the free carrier contribution to the complex refractive index^20^.

Indeed, also in our case, the transmittance curves in Fig. 1a can be modeled by a frequency dependent Drude like background and by a Lorentzian phonon resonance at ν_0_ = ω_0,L_/2π = 62 cm^−1^ using the dielectric function: $\varepsilon \left(\omega \right)={\varepsilon }_{\infty }+ \frac{{\omega }_{p, D}}{{\omega }^{2}-i{\Gamma }_{D}\omega }+\frac{{\omega }_{p, L}}{{{{\omega }^{2}}_{0L}-\omega }^{2}-i{\Gamma }_{L}\omega }$. The first term ${\varepsilon }_{\infty }$ is the dielectric function at high frequency, the second and third are the Drude and phonon contributions, respectively (see SI for details). Employing a model for the transmittance^21^ in which multiple reflections in the sample can be taken into account, the plasma frequency ω_p,D_ and the Drude damping constant Γ_D_ can be obtained by a fitting procedure (see supplementary information)_._ In the transmittance curves in Fig. 1a, the plasma frequency ω_p,D_ controls the total transmittance value and Γ_D_ the transmittance slope increase over the considered bandwidth. At room temperature, the value of ω_p,D_ / 2π = 7,305 cm^−1^ is remarkably close to the value determined from Hall effect data^19^ in bulk material. Moreover, ω_p,D_ is related to the free carrier concentration and their effective mass m^*^ via ${\omega }_{p,D}^{2}=\frac{N{e}^{2}}{{\varepsilon }_{0}{m}^{*}}$. Inserting the value for ω_p,D_ obtained from our fit (SI, Fig. S1), and using the measured N obtained within the Hall measurements (N = 1.3^.^10^20^ cm^−3^ of holes at 300 K, see Fig. 1e), an effective mass of m^*^ = 0.19⋅m_e_ is obtained and it compares well with values determined for bulk material^22^ (0.18⋅m_e_). Concerning the value of Γ_D_ = 205 cm^−1^ (300 K), it is only 8% smaller than the value for the bulk case and this might be related to a slightly different stoichiometry or to some structural difference between bulk and thin film structure. The temperature dependent fits of the transmittance data in Fig. 1a indicate an increase in ω_p,D_ by 20% at 5 K compared to the 300 K case (see SI for details). Considered that ${\omega }_{p,D}^{2}\sim N$, the finding is consistent with the measured increase of carrier density upon decreasing temperature (Fig. 1e).

In contrast to the commonly expected behavior, the frequency of the IR-active E_u_ phonon mode decreases when the temperature is lowered. Commonly, phonon frequencies decrease (soften) with raising temperature due to the increase in interatomic separations resulting from the thermal lattice expansion^23^. In fact, this conventional behavior is observed for the Raman-active phonon modes, as demonstrated in Fig. 1d by Raman spectra measured in the same temperature range. All Raman-active phonon modes exhibit a shift towards lower frequencies (softening), as expected in case of thermal lattice expansion^23^. From this result we can also exclude that the softening of the IR-active phonon mode is dominated by temperature-dependent strain induced by the mismatch between the thermal expansion of the silicon substrate and that of the Sb_2_Te_3_ film. Hence, only the E_u_-type IR-active mode shows the peculiar behavior upon T change–softening upon temperature decrease/hardening upon temperature increase–. This, and the fact that an energy shift reported in literature for similar materials (see below) is way smaller than 10 cm^−1^ as found in our case, suggests that the specific mode (E_u_) under investigation is strongly affected by anharmonicities^32,34^.

Shifts of soft phonon modes which usually cover a comparably large range of frequencies have been reported in literature and are usually attributed to low-temperature phase transitions. The most common ones are martensitic transitions or ferroelectric transitions^24,25^. Nevertheless both can be excluded since Sb_2_Te_3_ is known to be not ferroelectric^26^. Furthermore, Raman measurements performed in the same temperature range (see Fig. 1d) do not exhibit a qualitative change indicating a low-temperature phase transition. A further possible explanation could be the coupling of the phonon mode with the electronic system, which is treated as anharmonic contribution to the phonon self-energy. In semiconductors, the most general form of interaction between electronic excitations and phonons is the so-called deformation-potential mechanism^27^ treated as anharmonic contribution of the harmonic lattice dynamics phonon together with other sources of anharmonicities such as thermal expansion effects and phonon decay into multiple phonons. The thermal expansion is referred as the quasi-harmonic approximation and its contribution to frequency shifts is very small at low T. In literature there are many examples of doping induced renormalizations of the frequency as well as the lifetime of the phonons at low T^27^. Shifts to lower frequencies of phonon lines in Raman spectra and a broadening of these lines were reported in n- and p-type Si and p-type Ge^28,29^. Similar results have also been found for heavily doped GaAs^30^. Furthermore, several examples for transition-metal chalcogenides^31,32^ and other narrow band gap semiconductors^33^ have been given. In our case, the measured hole concentration changes in the unintentionally p-doped Sb_2_Te_3_ in Fig. 1e) and its corresponding peculiar E_u_ peak behavior upon decreasing T. Such behavior points toward strong anharmonicities contribution on the phonon self-energy. Those results compare well with similar topological insulators systems such as Bi_2_Te_3_ and Bi_2_Se_3_^32,34^, in which the anharmonic contributions are attributed to coupling between surface phonons and surface plasmon originated by the bulk free carriers^32^.

Besides the temperature-dependent frequency shift, the E_u_ phonon mode exhibits also a pronounced broadening with increasing temperature, as shown in Fig. 1c. The phonon line width Γ_L_, extracted by fitting the transmittance data in Fig. 1a utilizing the model for the dielectric function (third term in the formula, see SI), decreases with decreasing temperature T, changing from Γ_L_ = 12.6 cm^−1^ (300 K) to Γ_L0_ = 6.7 cm^−1^ (5 K), where Γ_L0_ is the line width for T → 0 K. The trends for Γ_*L*_ and τ are qualitatively in line with what observed for optical phonons in most semiconductors^35^ but, the lifetime of a phonon τ is limited by interactions with boundaries, defects, free carriers, and other phonons. Decoupling of the multiple anharmonic contributions to the phonon self-energy is not trivial, especially for highly doped materials such as in our case, where strong influence of the band gap and carrier densities starts to play a role.

To understand the observed temperature dependence of the peak width Γ_L_, the experimental data from Fig. 1c were fitted by the relation ${\Gamma }_{L}(T)={\Gamma }_{1}{+\Gamma }_{0}\left(1+{2/(e}^{\frac{\hslash {\omega }_{0}}{2kT}}-1)\right)$ within the model of Klemens^36^, who assumed a phonon decay via the so-called three phonon decay channel, where the phonon at ω_0_ decays into two phonons of ± ω_0_/2. Following such a symmetrical decay like in Beechem et al.^37^, one finds  ${\Gamma }_{1}$ = 6.02 cm^−1^, ${\Gamma }_{o}$ = 0.54 cm^−1^ as free fit parameters with ${\Gamma }_{1}+ {\Gamma }_{0}$=${\Gamma }_{L}$(0) and ω_0_ = 2πν_0_ (ν_0_ = 61.7 cm^−1^) the resonant frequency of the E_u_ phonon mode for T → 0, as derived from Fig. 1b. As compared in Fig. 1c, the simple model and the measured values of ${\Gamma }_{L}$ show excellent agreement at low temperatures but become gradually different as temperature approaches room temperature values. At higher temperatures > 125 K, the Fermi–Dirac occupation of charge carriers and Boltzmann occupation of phonon states as well as thermal expansion effects along with a gap change^18^ dominate the slope rather than the pure microscopic decay mechanism.

### Part 2: Time resolved measurement

The phonon-carrier interaction has been also investigated within a time-resolved experiment employing the THz probe-fs-laser pump setup depicted in Fig. 2a (see “Methods” and SI Fig. S3).

Figure 2c shows the temporal evolution of THz transmittance for Sb_2_Te_3_ on Si (111) in the spectral range between 10 and 100 cm^−1^ at low (0.6 mJ/cm^2^) and high (3 mJ/cm^2^) laser fluences. Let’s recall that a pump-induced decrease of the THz transmission can be phenomenologically attributed to an increase of the conductivity in the material due to generation of free carriers. Indeed, both signal evolutions show a resolution limited drop (~ 0.7 ps) attributed to the photo-injection of carriers. The drop is deeper for higher pump fluences, indication of higher carrier density production in respect to the low fluence case, as expected. For the high fluence case, the signal recovery displays an exponential increase with a fast time constant of τ_f2_ = 4.2 ± 0.4 ps followed by a second recovery with slower time constant of τ_s_ = 169 ± 17 ps (see Fig. 2b). At low fluence instead, the signal recovery can be fitted with only one fast time constant of τ_f1_ = 5.1 ± 0.5 ps (~ 18% slower decay compared to τ_f2_ of high fluence case). This suggests that the higher the density of photoexcited carriers and their energy (larger width in the frequency domain), the faster the phonon damping (τ_f1_ > τ_f2_ in the time domain), as also found for silicon^38^.

Within the fast decay time (τ_f1_ and τ_f2_), it is reasonable to assume an interplay between different fast carrier thermalization and recombination pathways such as phonon emission and recombination via traps and defects. The slower recovery might be linked to the presence of ordered vacancy layers. The latter could represent another recombination channel for photo-excited carriers that cannot recombine through alternative faster pathways^5^. Therefore, here we focus on spectrally resolved ultrafast response in the low fluence case only. Spectrally resolved snapshots (resolution of 1 cm^−1^) at given delay time can also be measured in the spectral range between 25 and 120 cm^−1^ under specific operation of the setup depicted in Fig. 2 (see “Methods”).

**Figure 3:**
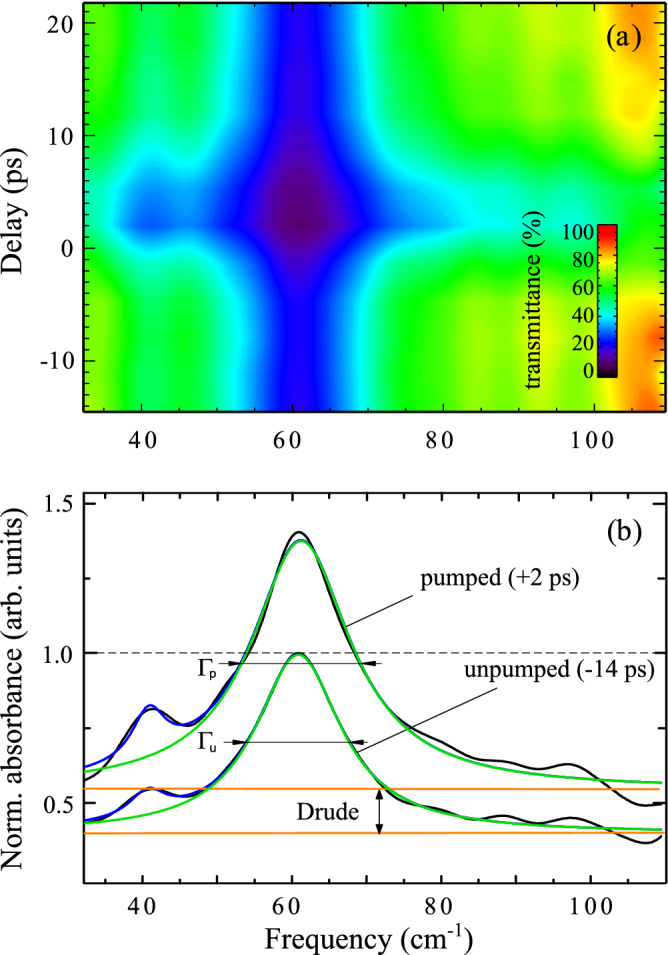
**(a)** Color-coded 2D time–frequency map of the relative transmittance change in Sb_2_Te_3_ films upon excitation with fs-laser pulses of 100 fs at 1.55 eV energy and at 0.1 mJ/cm^−2^ fluence. **(b)** Result of the Lorentz fits (blue-total, green-phonon) of the transient linear normalized absorbance peak in the 80 nm Sb_2_Te_3_ epitaxial film. The total fit curve (blue) also takes into account the satellite peak at 40 cm^−1^. The peak at 62 cm^−1^ (E_u_) shows both, a transient intensity increases as well as a change of its pumped spectral width Γ_p_ 15% larger upon 1.55 eV pumping than the unpumped width Γ_u_. A broad band offset growth beneath the phonon line (orange lines) indicates the Drude free carrier part of the dielectric function. The absorbance is normalized to the spectra taken at −14 ps delay before time zero.

A full color-coded plot of the transmittance spectra *T*(t) = I/I_0_, where I is the detected signal and I_0_ is the incident THz signal is shown in Fig. 3a. In Fig. 3b instead, we show selected spectra of the absorbance A = log [1/*T*(t)] (normalized to the spectra taken at −14 ps delay before time zero) and corresponding Lorentz fits from before (−14 ps) and after (+2 ps) time-zero, in other words, the THz probe before and after the pump excitation (“unpumped” and “pumped” labels, respectively). The color-coded plot of the transmittance in Fig. 3a reveals some interesting features: (1) the total broad band transmittance significantly drops in a transient state immediately after laser excitation (−43% transmittance due to an increase of absorbance) and the THz signal recovers within ~ 20 ps; (2) the observed main transmittance dip at ~ 62 cm^−1^ corresponds to the E_u_ phonon resonance discussed within the static temperature dependent experiment. The satellite dip at ~ 40 cm^−1^ was previously reported and attributed to the E_g_(1) phonon mode^39^. In the following we focus only on the main resonance at ~ 62 cm^−1^ because no dynamic change at our available resolution could be observed for the satellite feature; (3) a width change within a time interval < 20 ps is measured, but no frequency shift is observed. A more quantitative analysis has been done by fitting the absorption peaks in Fig. 3b within the framework of a Drude-Lorentz model (see “Methods” and SI for fitting details).

**Figure 4:**
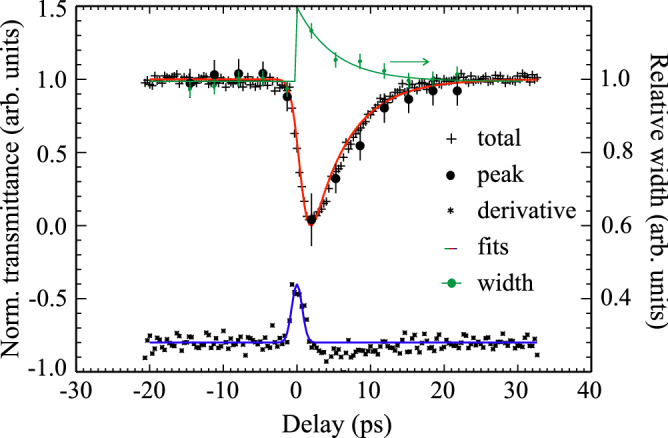
Normalized transient THz transmittance delay traces from the total signal ( +) and the peak transmittance dip (black dots) of the phonon resonance compared to single exponential fits (red line) with a recovery constant of τ = 5.1 ps. Green dots are width measurements and orange diamonds the free carrier offset change as derived from Lorentz fits to the phonon peak and its spectral background. The green line is a single exponential fit to width data using the same τ as for the red line. The derivative of the total trace (black stars) is compared to a Gaussian as derived from the fits (blue line) indicating the time resolution limit.

To extract the E_u_ phonon parameters from our dynamic data for the pumped (A_p_ and Γ_p_) and unpumped (A_u_ and Γ_u_) cases, we disentangled the time dependent Drude-background intensity and we compared its temporal intensity evolution in Fig. 4 to the total and the phonon related absorption contributions, as well as to the peak width change Γ_p_/Γ_u_ determined from the fits plotted there. The main result of that comparison is that both, the phonon recovery as well as the dynamics of the Drude-like free carriers, are damped within the same time constant of τ  ~ 5 ps.

The phonon lifetime corresponding to the phonon damping constant Γ_L_ shown in Fig. 1c varies between 0.8 ps > τ =$\hslash$ / Γ_L_ > 0.45 ps according to Heisenberg's uncertainty relation (see SI, Fig. S2). The room-temperature value τ  ~ 0.45 ps is, thus, one order of magnitude smaller than what is observed by our time-resolved experiments. This finding and the same decay time for both carrier and lattice dynamics might be explained by a high correlation of the two systems, as also emerged for the time dependence experiment. Moreover, the time resolved experiment suggests that energy thermalization of laser-excited carriers via the emission of phonons is the main mechanisms on the fast time scale of ~ 5 ps. Within this simulation we also cross-checked (see SI, Figs. S4-6) that after excitation at low fluence, laser induced lattice strain in the first 20 ps has negligible influence on the observed fast dynamics (< < 1%)^40^.

If we recall the static experiment in the previous section, we learned that both, peak width and frequency strongly decreased upon temperature decrease between 300 and 5 K. In the time resolved experiment instead, the phonon peak width shows a transient rise (15% between pumped and unpumped shown in Fig. 3b) but no frequency shift is detected. A reason could be that we are performing the experiment at T > 300 K, a temperature at which the phonon frequency shift might be less significant and saturate, as also observed in the static case at ~ 300 K (see Fig. 1b, c). Note that within the framework of lattice temperature simulations (see SI, Figs. S4-S6) it is calculated that the temperature increases of the total system at 300 K, induced by the pump laser, is of only 10 K. Moreover, the total absence of any frequency shift might also be attributed to the fact that the optical excitation of Sb_2_Te_3_ at 800 nm (1.55 eV) generates free electron–hole pairs by interband transitions. The effect of hole or electron doping on the phonon shift would be in opposite directions^41^, giving an average cancelation of the peak shift.

## Conclusions

In summary, the combination of static and time resolved FIR, Raman and THz spectroscopy techniques have been employed to investigate electron–phonon coupling in epitaxial Sb_2_T_3_ at thermal equilibrium as well as in transient states after femtosecond laser excitation.

First, static THz spectroscopy is used to probe an in-plane phonon mode at thermal equilibrium upon changing temperature. The absorption feature at ~ 62 cm^−1^ is assigned to the IR-active E_u_ phonon mode, which shows a peculiar softening of 10 cm^−1^ wavenumbers upon temperature decrease. Comparison with Raman measurements shows that such behavior is only observed for this phonon mode suggesting how strongly it is affected by anharmonicities as found in similar material systems^41^. At low temperatures the phonon linewidth Γ_L_ follows the microscopic two phonon decay model^36,37^ but deviates from that at temperatures > 125 K, where Fermi–Dirac occupation of charge carriers and Boltzmann occupation of phonon states as well as thermal expansion effects along with a gap change^18^ come gradually into play.

Then, by ultrafast time resolved measurements we have demonstrated that laser excitation of the films at very low fluences leads to strong changes of both, the same phonon mode and free carriers. The recoveries after the laser excitation of the two systems are decoupled in the analysis and it was found that both systems relax on the same time scale of ~ 5 ps, a factor of 10 slower than expected from the time-bandwidth limit of the phonon resonance. This again points toward a strong coupling of carrier and lattice through scattering of photoexcited carriers with the IR active phonon mode. The results also show that THz spectroscopy techniques are powerful tools to investigate Sb_2_Te_3_ properties that can be exploited for applications such as in photonic and electronic memories and processors, in THz detection and thermoelectric devices. These findings combined to material engineering could illuminate future avenues for device improvements.

## Methods

### MBE growth

A series of Sb_2_Te_3_ films, unintentionally doped, were deposited by MBE on a highly resistive (5 kΩcm^−1^) crystalline Si (111) − (√3 × √3)R30-Sb surface with a thickness ranging between 30 and 80 nm^42^. The samples were capped with 35 nm of ZnS–SiO_2_ by sputtering to prevent oxidation of the films.

### Raman measurement

Raman spectra were acquired exciting samples with the 632.8 nm line of a He–Ne laser and the scattered light was analyzed using a single spectrograph (Horiba/Jobin–Yvon LabRam HR Evolution) equipped with an LN_2_-cooled charge-coupled device detector. The spectra were recorded in backscattering geometry from the sample surface. For the low temperature measurements, a continuous-flow microscope cryostat (Cryovac KONTI) was employed during Raman spectra acquisition.

### Static far infrared measurements

Measurements in the far-infrared regime were carried out under vacuum conditions in transmission geometry using a high-resolution Fourier transform infrared spectrometer (BRUKER IFS 125HR) of the THz beamline at Helmholtz-Zentrum Berlin (HZB, BESSY II)^43^. The spectral range in the presented experiments covered wavenumbers between 20 and 120 cm^−1^ (i.e., frequencies from 0.6 to ~ 3.6 THz) and was limited by the selected source(s), the 6 µm multilayer-Mylar beamsplitter and the detector, a 4.2 K Si-Bolometer from Infrared Labs. Static measurements were also repeated using Coherent Synchrotron radiation (CSR) from the slicing source (see “Methods” below) to prepare time-resolved studies. Low temperature measurements were performed by employing an optical LHe-cryostat Oxford Optistat CF2 equipped with Quartz windows and installed in-vacuum inside the sample compartment of the IFS125 HR in transmission geometry.

### Ultrafast THz-Fourier transform spectroscopy (THz-FTS)

Optical pump-THz spectral probe measurements were carried out using the laser-slicing method at the BESSY II storage ring^44,45^. Here single cycle THz pulses are generated by laser-energy modulation of relativistic electrons at 6 kHz repetition rate, extracted from a dipole source in the ring and merged again with the naturally synchronized laser pulse about 17 m after the interaction in the sample compartment of the Fourier transform spectrometer IFS125 HR^43^. As depicted in Fig. 1a, a part of the slicing laser (few 100 µJ pulse energy, 100 fs length, λ = 800 nm) is used to excite the sample (absorption in film see SI Fig. S3) while the naturally synchronized THz pulse probes the relative transmittance change in the Sb_2_Te_3_ film. The diameter of the THz pulse was set by apertures to ~ 1.5 mm diameter adopting the laser spot to somewhat larger in size than the THz probe allowing for fluence values up to few mJ/cm^-2^ on the sample. The angle of incidence between laser and THz pulse was about 20° limiting the time-resolution to ~ 0.7 ps FWHM by geometrical pulse elongation. Time resolved spectra were acquired performing FTS with a 4.2 K Si-bolometer as detector and gated detection at 6 kHz using the UHFLI^46^ lock-in device and its digital boxcar function. The laser is modulated with a 3 kHz mechanical chopper as in Fig. 2a to probe the pumped and un-pumped cases separately. Time resolution is achieved by measuring spectra at different arrival time delays between fs-laser and THz probe pulse as controlled by a mechanical delay stage equipped with optical encoders. The spectral bandwidth of the THz pulse was thoroughly obtained in Ref.^10^, it peaks at about 2 THz ranging from 0.3 to 3 THz (~ 10 to 100 cm^−1^). All THz transmittance spectra were determined using the same optical geometry, but without Sb_2_Te_3_, film as a reference. The laser fluences are determined as described in SI.

## Supplementary information

Supplementary Information.
