# Supplementary material for: Phonon anharmonicities and ultrafast dynamics in epitaxial Sb2Te3
Source: Sci Rep. 2020 Jul 31;10:12962. doi: 10.1038/s41598-020-69663-y (PMC7395099; doi:10.1038/s41598-020-69663-y)
Supplement: Supplementary file 1 — Supplementary Information. [file 41598_2020_69663_MOESM1_ESM.docx]

**Supplementary Information**

**Phonon anharmonicities and ultrafast dynamics in epitaxial Sb_2_Te_3_**

V. Bragaglia^1,2*^, M. Ramsteiner^1^, D. Schick^3,4^, J. E. Boschker^1^, R. Mitzner^3^, R. Calarco^1,5**^ and K. Holldack^3***^

*^1^ Paul-Drude-Institut für Festkörperelektronik, Hausvogteiplatz 5-7, 10117 Berlin, Germany*

*^2^ Currently at IBM Research – Zürich, Säumerstrasse 4, CH–8803 Rüschlikon, Switzerland*

*^3^ Helmholtz-Zentrum Berlin für Materialien und Energie GmbH, Albert-Einstein-Str. 15, D 12489 Berlin, Germany*

*^4^ Max-Born-Institut für Nichtlineare Optik und Kurzzeitspektroskopie, Max-Born-Straße 2A, 12489 Berlin, Germany*

*^5^Istituto per la Microelettronica e Microsistemi (IMM), Consiglio Nazionale delle Ricerche, Via del Fosso del Cavaliere 100, 00133, Rome, Italy*

*vbr@zurich.ibm.com, **raffaella.calarco@cnr.it, ***karsten.holldack@helmholtz-berlin.de

**Drude-Lorentzian fit to static and dynamic data**

We simulated our transmittance data using a simple Drude-Lorentzian model based on the complex dielectric function:

$\varepsilon\left( \omega\right)=\varepsilon_{\infty}+ \sum_{j} \frac{\omega_{pj}}{{\omega^{2}}_{0j}-\omega^{2}-i\Gamma_{j}\omega}$, (1)

in which ω_pj_ is the plasma frequency, ω_0j_ is the Lorentz resonance frequency and j = D, L, with D and L indicating the Drude or Lorentzian contributions, respectively. In case of the Drude term, $\omega$_0D_ = 0 and the Drude scattering can be modelled as Lorentzian resonance at zero wavenumbers. With only one Lorentz resonator in our spectral range, Formula 1 reduces to:

$\varepsilon\left( \omega\right)=\varepsilon_{\infty}+ \frac{\omega_{pD}}{\omega^{2}-i\Gamma_{D}\omega}+\frac{\omega_{pL}}{{{\omega^{2}}_{0L}-\omega}^{2}-i\Gamma_{L}\omega}$,. (2)

As starting point for the fit of the transmittance data in Figure 1 we used the Drude values (ω_pD_ and Γ_D_) determined in Ref.^2^ for bulk Sb_2_Te while the Lorentz values have been directly extracted from the peak position of the experimental data. In addition, we compared the Drude values of Ref.^2^ with the Drude plasma frequency determined from our own Mid-IR total Reflectance measurement on a bulk powder pellet of Sb_2_Te_3_ (Sigma-Aldrich). Here, a fit to the plasma edge yielded 7600 cm^-1^ at a corresponding scattering rate of 205 cm^-1^, very close to single crystal bulk data from Manson et al.^2^

Using these parameters as starting values for the Drude-Lorentz fit and keeping Drude and Lorentz values as free parameters, we obtained the fitting curve shown in Figure S1 and the corresponding parameter set as reported in table S1. Error bars of the fit are of the order of 10 % for scattering rates and 0.2 cm^-1^ for the peak position.


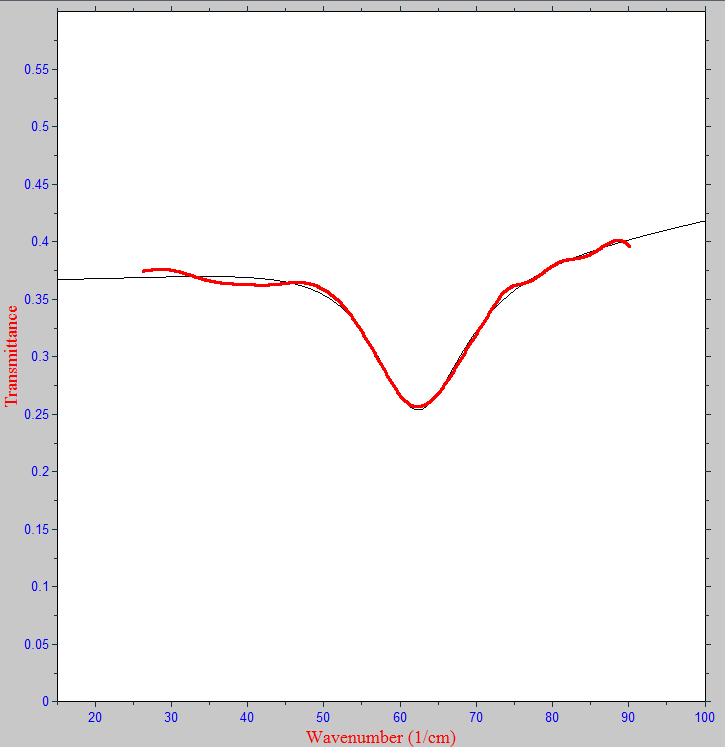


Figure S1: Sb_2_Te_3_ film transmittance T = I_sample_(ν)/I_Si_(ν) at room temperature (red) and best fit result (grey) using a fitting code^1^  which takes multiple reflections into account using the fit parameters according to the Drude-Lorentz model of Formula 1. The fit results are summarized in Table S1

| **Model Temp.** | **ω_p_ /2π (cm^-1^)** | **ω_0_ /2π (cm^-1^)** | **Γj (cm^-1^)** |
| --- | --- | --- | --- |
| **Drude, j=D 300 K** | 7305 | 0 | 205 |
| **Lorentz, j=L 300 K** | 1347 | 62.3 | 12.2 |

Table S1: Fitting results of 300 K transmission data using the Drude-Lorentz dielectric function (2) and the fitting code from Ref.^1^.

**Lorentz limit and natural linewidth**


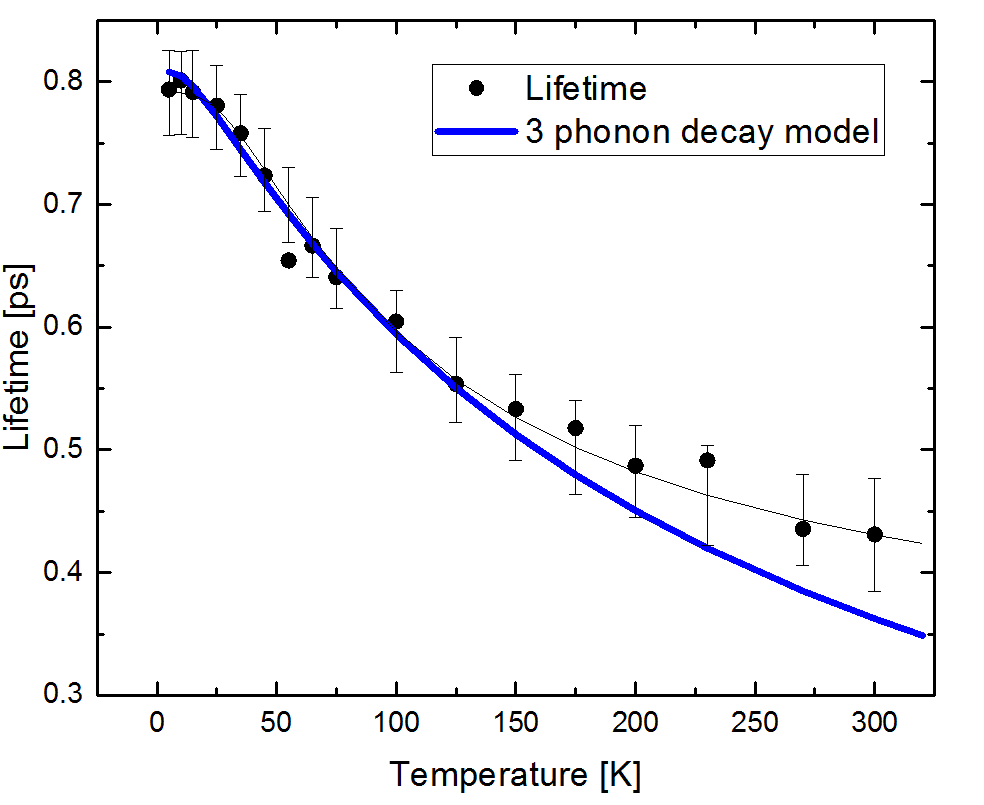


Figure S2: Measured phonon lifetime derived from the Lorentz-Limit (black dots) and the lifetime trend expected from the symmetric 3 phonon decay model according to Ref^3^ (blue line).

**Pump laser issues**

The power absorption [%.nm^-1^] of the 800 nm fs-laser (COHERENT, LEGEND Elite Pro) in a multilayer system composed by 35 nm ZnS-SiO_2_ cap layer, 80 nm Sb_2_Te_3_ and 500 µm Si is depicted in Fig. S1. There is only 0.5% power deposited in the bulk Si which is THz transparent. The cap-layer is also transparent for 800 nm fs-laser.

**
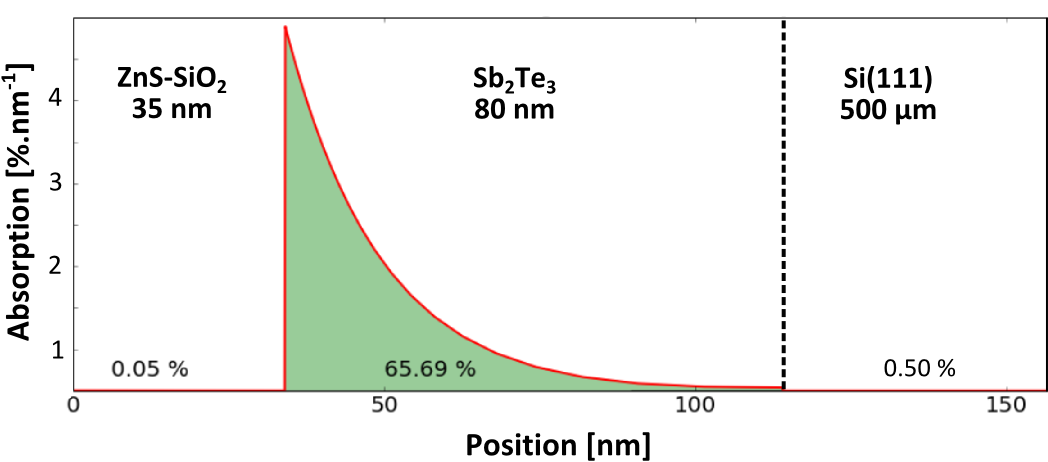
**

Figure S3: Absorption fractions of the initial laser fluence along the depth of the sample. The main part is deposited into the 80 nm Sb_2_Te_3_ film and only 0.5 % into the Si substrate beneath.

**Lattice temperature and strain**


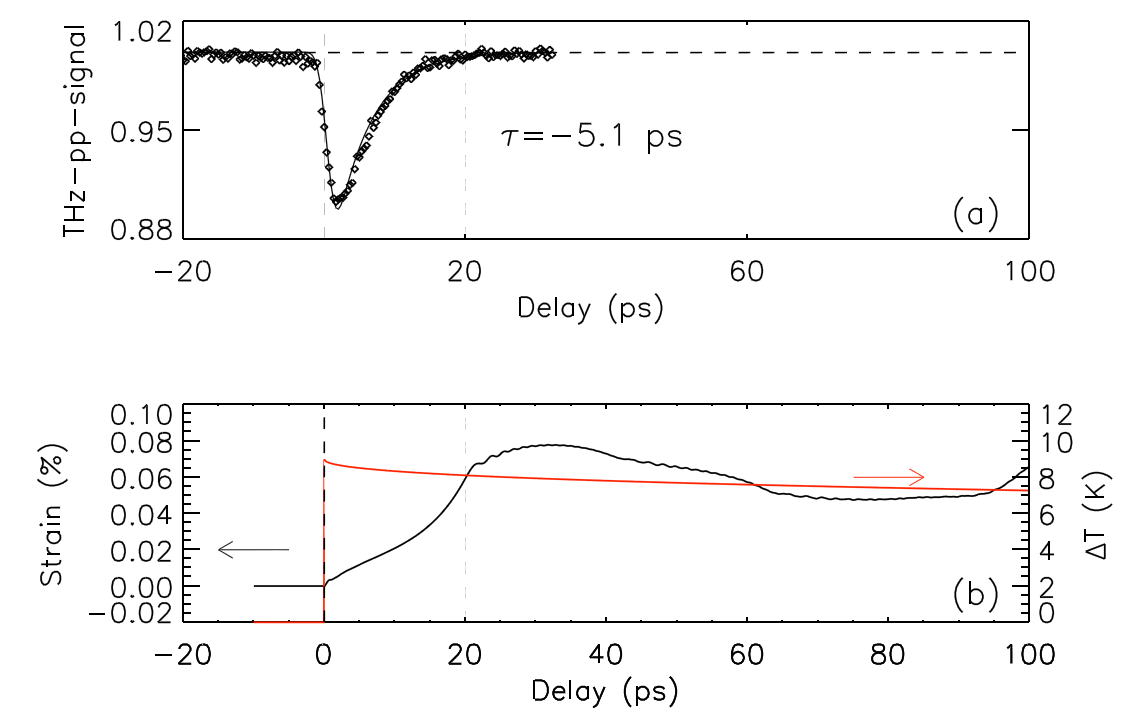


Figure S4: a) Decay and recovery of the total THZ signal for the low fluence case and exponential fit. b): Simulated lattice strain (left axis) and temperature rise of the lattice calculated in Sb_2_Te_3_ for the low fluence case. The thermal lattice response is as slow as the measured slow time constant τs = 169 ps in Fig. 2 of the main text.^4^


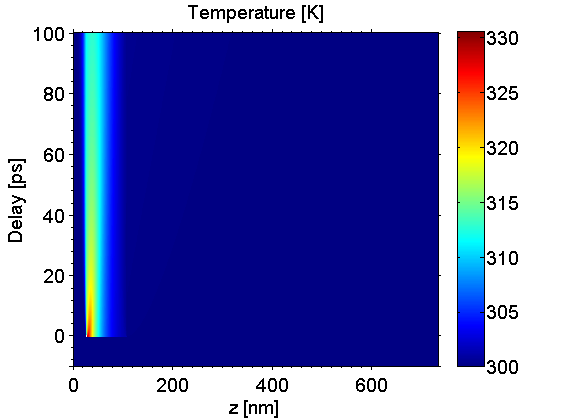


Figure S5: 2D plot of the laser induced temperature rise in the Sb_2_Te_3_ layer after laser excitation for the case of 0.1 mJ/cm^-2^ laser fluence. Main heat is generated in the Sb_2_Te_3_ layer decaying with τ_s_.^4^


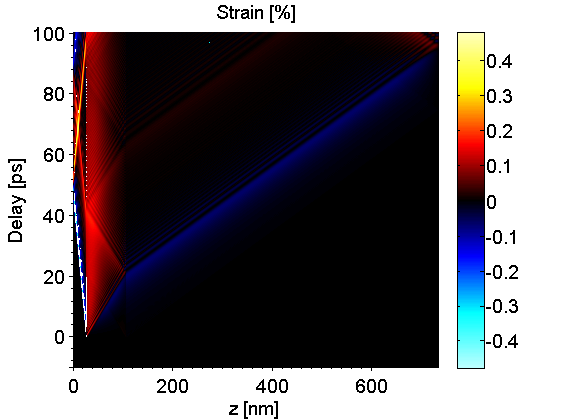


Figure S6: 2D Strain propagation simulation along the layer structure. The interference pattern appears due to multiple reflections bouncing back and forth with sound velocity in the Sb_2_Te_3_ layer. The blue part indicates some residual strain propagating in the Si substrate.^4^

**Determining the laser fluence**

In order to obtain the laser fluence, we have imaged the laser spot by using a laser beam profiler (Fig. S7) and then, known the laser power used to pump the sample and the laser repetition rate (6 kHz), we used the conversion table shown in Fig. S8.

**
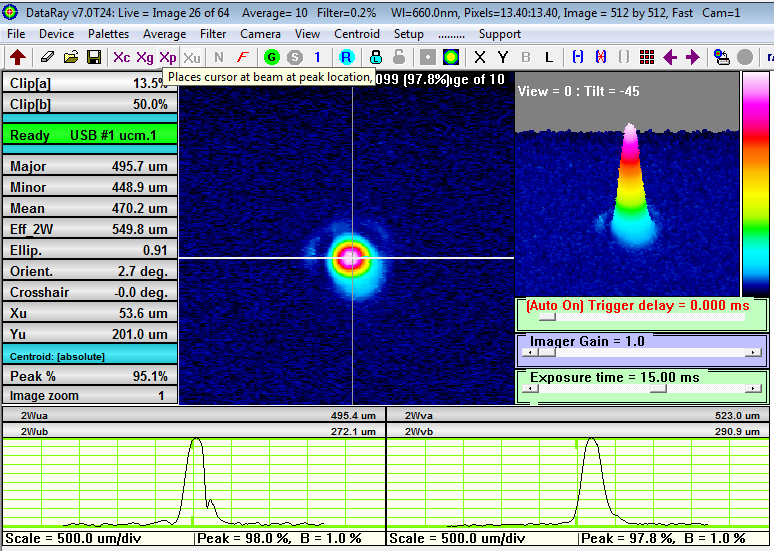
**

Figure S7: Spot Size measurement of the laser spot at the sample position

**
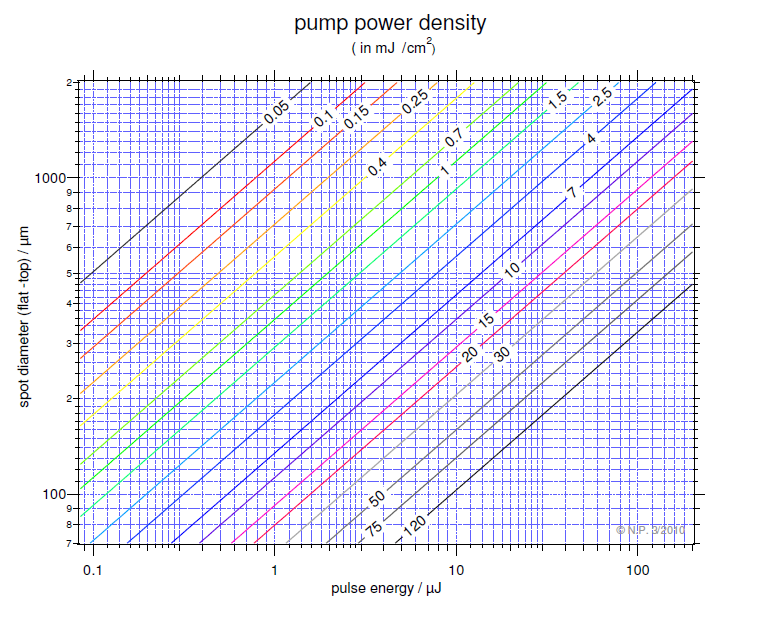
**

Figure S8: Conversion table between pulse energy and fluence

**References:**

1. Optical simulation program *RefFIT* by A. Kuzmenko, [*https://sites.google.com/site/reffitprogram/home*](https://sites.google.com/site/reffitprogram/home)*.*
2. J. Manson, A. Madubuonu, D. A. Crandles, C. Uher, P. Lostak, Infrared spectroscopy of Cr- and V-doped Sb_2_Te_3_: Dilute magnetic semiconductors, *Phys. Rev. B* **90**, 205205 (2014).
3. P. G. Klemens, Anharmonic Decay of Optical Phonons, *Phys. Rev.* *B* **148**, 845 (1966).
4. D. Schick, A. Bojahr, M. Herzog, R. Shayduk, C. von Korff-Schmising, and M. Bargheer, Udkm1Dsim - A simulation toolkit for 1D ultrafast dynamics in condensed matter, *Comput. Phys. Commun.* **185**, 651 (2014).
